# Socially Important Faces Are Processed Preferentially to Other Familiar and Unfamiliar Faces in a Priming Task across a Range of Viewpoints

**DOI:** 10.1371/journal.pone.0156350

**Published:** 2016-05-24

**Authors:** Helen Keyes, Catherine Zalicks

**Affiliations:** Department of Psychology, Anglia Ruskin University, Cambridge, Cambridgeshire, United Kingdom; Vanderbilt University, UNITED STATES

## Abstract

Using a priming paradigm, we investigate whether socially important faces are processed preferentially compared to other familiar and unfamiliar faces, and whether any such effects are affected by changes in viewpoint. Participants were primed with frontal images of personally familiar, famous or unfamiliar faces, and responded to target images of congruent or incongruent identity, presented in frontal, three quarter or profile views. We report that participants responded significantly faster to socially important faces (a friend’s face) compared to other highly familiar (famous) faces or unfamiliar faces. Crucially, responses to famous and unfamiliar faces did not differ. This suggests that, when presented in the context of a socially important stimulus, socially unimportant familiar faces (famous faces) are treated in a similar manner to unfamiliar faces. This effect was not tied to viewpoint, and priming did not affect socially important face processing differently to other faces.

## Introduction

Attention biases in face perception have long been a topic of interest. Recently, focus has turned to whether there are special attention biases related to socially important stimuli. Indeed, Keyes and Dlugokencka [1] report that socially important personally familiar faces can automatically recruit our attention when presented outside the direct focus of attention. In that paper, we showed that when a friend’s face is presented peripherally to a central task, it can cause an automatic distraction. This was not the case for unfamiliar faces, or for a participant’s own face. We inferred a “social importance” effect, whereby we may be tuned to pick out and pay attention to socially relevant faces even when presented outside the focus of attention—for example, in a crowd. Others have also begun to ask whether socially important stimuli selectively recruit attention. For example, Devue and Brédart [2] showed that personally familiar faces (a participant’s own face and a friend’s face) selectively capture attention compared with unfamiliar faces. However, no studies to date have attempted to isolate the effect of personal familiarity as an attentional cue, dissociating the effects of social importance from the effects of mere familiarity.

In order to examine the functional and neural substrates of social attention, an important question to ask is what we define as a “socially important” stimulus. For the most part, research on the mechanisms underlying the recruitment of attention in face recognition has tested for familiarity effects using famous face stimuli (e.g. [3–6]). In recent years, the use of famous faces to test for familiarity effects has been criticised. Carbon [7] demonstrated that in some instances famous face processing may be tied to iconic or pictorial representations of those faces, rather than facial representations per se. Participants were presented with original, digitally modified and uncommonly seen images of famous and personally familiar faces, along with unfamiliar faces, and were requested to name the person in the image. Carbon hypothesised that if famous faces were represented in an iconic manner (such as the iconic images of Che Guevara or Marilyn Monroe), their representations in memory would be rigid and limited, and so image distortion or uncommon images of that person should impair recognition. This should not be the case for personally familiar faces, for which we have experience in viewing over a variety of conditions, and thus our representations of those faces should be more robust to change. Indeed, Carbon reported that recognition performance for famous faces decreased significantly and substantially when these faces are digitally manipulated or uncommon versions are presented. In contrast, personally familiar face recognition rates did not differ substantially across testing conditions. Carbon concluded that the ‘face expertise’ we possess for personally familiar faces cannot be achieved through even extensive exposure to iconic images. In the current paper, we focus on whether socially important (personally familiar) faces may continue to have a processing advantage over familiar famous faces when these famous faces are not tied to iconic representations. That is, we use famous faces that participants will be familiar with viewing from a variety of viewpoints and across a variety of situations (film stars and other celebrities whose faces are typically viewed dynamically and in motion).

Herzmann and colleagues also demonstrated differences in famous and personally familiar face processing [8]. They analysed amplitude at the N250r, an ERP component noted for being particularly large for familiar relative to unfamiliar faces (e.g. [9]), suggesting that it represents activation of facial representations stored in memory, or face recognition units [10]. Herzmann and colleagues observed that N250r activity in response to personally familiar faces was significantly larger than activity observed in response to famous faces, which in turn was larger than activity for unfamiliar face viewing. They interpret this finding as evidence that personally familiar faces may have stronger networks of representation than famous faces, which is in line with Tong and Nakayama’s [11] suggestion that personally familiar faces have particularly robust representation. Herzmann and colleagues also reported significantly larger skin conductance response—an index of emotional processing—for personally familiar faces, compared to either famous or unfamiliar faces (between which there was no difference). This heightened emotional response suggests a role for social importance in our perception of personally familiar faces.

Indeed, more researchers are suggesting that it is precisely the social importance of personally familiar faces which makes us process them differently than other familiar faces. An fMRI study showed increased activation in the medial temporal lobe when images of social relevant (family) faces were shown to participants, compared to famous faces [12] (see also [13]), and Gobbini and colleagues show increased activation in brain areas typically associated with “theory of mind” when participants viewed personally familiar faces, which were thought to elicit stronger emotional attachment, compared to either famous or unfamiliar faces [14]. Meanwhile, an MEG study found larger M170 amplitude in response to personally familiar faces—but not famous faces—compared to unfamiliar faces [15] (see [16] for EEG evidence). Very recently, Liccione and colleagues have called for the *phenomenological* importance of a face to be taken into account in face perception studies, suggesting that personally familiar faces may elicit the possibility of relational engagement, and thus may be processed preferentially to other types of familiar face [17].

### Aims

Recent work has suggested that we may process socially important familiar faces preferentially to famous faces [8], [16–17]. We investigate that here using a priming paradigm. As well as directly addressing the question of speed of processing differences for socially important and famous faces, a priming paradigm allows us to investigate the potential role of attentional effects in these differences. Both Keyes and Dlugokencka [1] and Devue and Brédart [2] report that socially important faces can selectively “grab” attention. Novel to this study, we ask: if we are primed to pay attention to a socially important stimulus (e.g. by the presentation of a personally familiar face), will the social relevance of the prime stimulus speed our processing of a related target stimulus, relative to priming with a socially unimportant stimulus (e.g. a famous face or an unfamiliar face)?

A secondary question of this research is whether social importance (personal familiarity with a face) will lead to greater viewpoint-independence when processing that face, due to a more established robust representation of these faces. Logie, Baddeley and Woodhead [18] showed that for unfamiliar faces, recognition performance decreased when viewpoint changed between learning and test phases. This suggests that, for unfamiliar faces at least, we rely heavily on pictorial or viewpoint dependent cues when recognising a face. We predict that a change in viewpoint between prime and target faces will affect unfamiliar faces more detrimentally than familiar faces [19–22], as familiar faces representations should contain more view-invariant information. We further investigate whether socially important faces (personally familiar faces) will suffer less from a change in viewpoint than will famous faces. This has not previously been examined, with other researchers either comparing famous with unfamiliar faces [19–21] or personally familiar with unfamiliar faces [22].

## Materials and Methods

### Pilot Study

In order to ensure that the famous faces used in the main experiment were likely to be well known to participants, a pilot study was carried out using 15 participants (7 female) who did not take part in the main experiment. These participants had a mean age of 22.7 years (SD = 3.7). Participants were shown a series of famous faces using a Powerpoint presentation. For each face, they were asked to write down the name of the person if they knew it, and to rate how familiar they were with that person’s face on a scale of 1–10, where 1 = *not familiar at all* and 10 = *extremely familiar*. Faces were matched for gender with the participant, and for each gender, only faces which could be named by all participants and which had an average familiarity rating greater than 8 were used as famous face stimuli for the main experiment.

### Participants

Forty participants (26 female) with a mean age of 22.5 years (SD = 5.3) volunteered to take part in the main study. Each participant was paired with a highly familiar same-sex and same-race friend whom they had known for at least one year, and whom they saw on a daily or almost daily basis. In most cases, this was a close friend from their undergraduate degree course, whom they had known for 2–3 years. The majority of the participants were recruited in pairs, where each person served as a friend for the other participant. Three males and three females who were unfamiliar to all participants consented to being photographed so that their images could serve as “unfamiliar” stimuli. Written informed consent was obtained from all participants prior to taking part in the study. The study received full ethical approval from the Faculty Research Ethics Panel (Science and Technology) at Anglia Ruskin University. Participants were paid £7 for their participation.

### Stimuli

For each participant, a close friend served as the model for their “friend” face stimuli, while a person unfamiliar to the participant served as the model to create their “unfamiliar” face stimuli. Famous faces were chosen using the process described in the Pilot Study section. To create the friend and unfamiliar stimuli, models were photographed under studio lighting conditions, posing with a neutral expression while looking directly at the 10.2 megapixel Nikon D80 camera (frontal view), at a 45° angle (three-quarter view), and at a 90° angle (profile view). For the famous face stimuli, three images each of a selection of famous people (Angelina Jolie, Emma Watson, Kristen Stewart, Daniel Radcliffe [actors], Beyoncé, Jay Z [singers], Wayne Rooney [sports star], and David Cameron [Prime Minister]) were taken from Google images. These images showed the famous person with a neutral expression, and comprised a frontal view, a three-quarter view and a profile view. For each participant, only one famous face identity was used in their famous face condition, and this face was gender- and race-matched with the participant. The quality of the famous face and friend/unfamiliar face photographs was comparable. Using Adobe Photoshop, images were converted to greyscale and matched for approximate luminance. An oval vignette (380 x 480 pixels) was applied to each facial image, ensuring that the jawline and hairline of each face were visible. Any background visible in the images was carefully removed using Adobe Photoshop and replaced with a light grey background. Images were viewed on a 17 inch screen of a Dell PC. Images subtended a viewing angle of 8.2 by 10.3 degrees when viewed from a distance of approximately 70 cm.

### Procedure

Prior to testing, participants were shown frontal view images of all three faces to be used in their trials (friend, famous, unfamiliar). The names identifying the faces were written below them on the screen. Participants were asked to look at the faces for as long as it took for them to be confidently able to identify each of the three faces. During this time, the participant also confirmed that the face being used for their “unfamiliar” condition was indeed unfamiliar to them, and that the friend face and famous face being used were indeed highly familiar to them.

**Fig 1:**
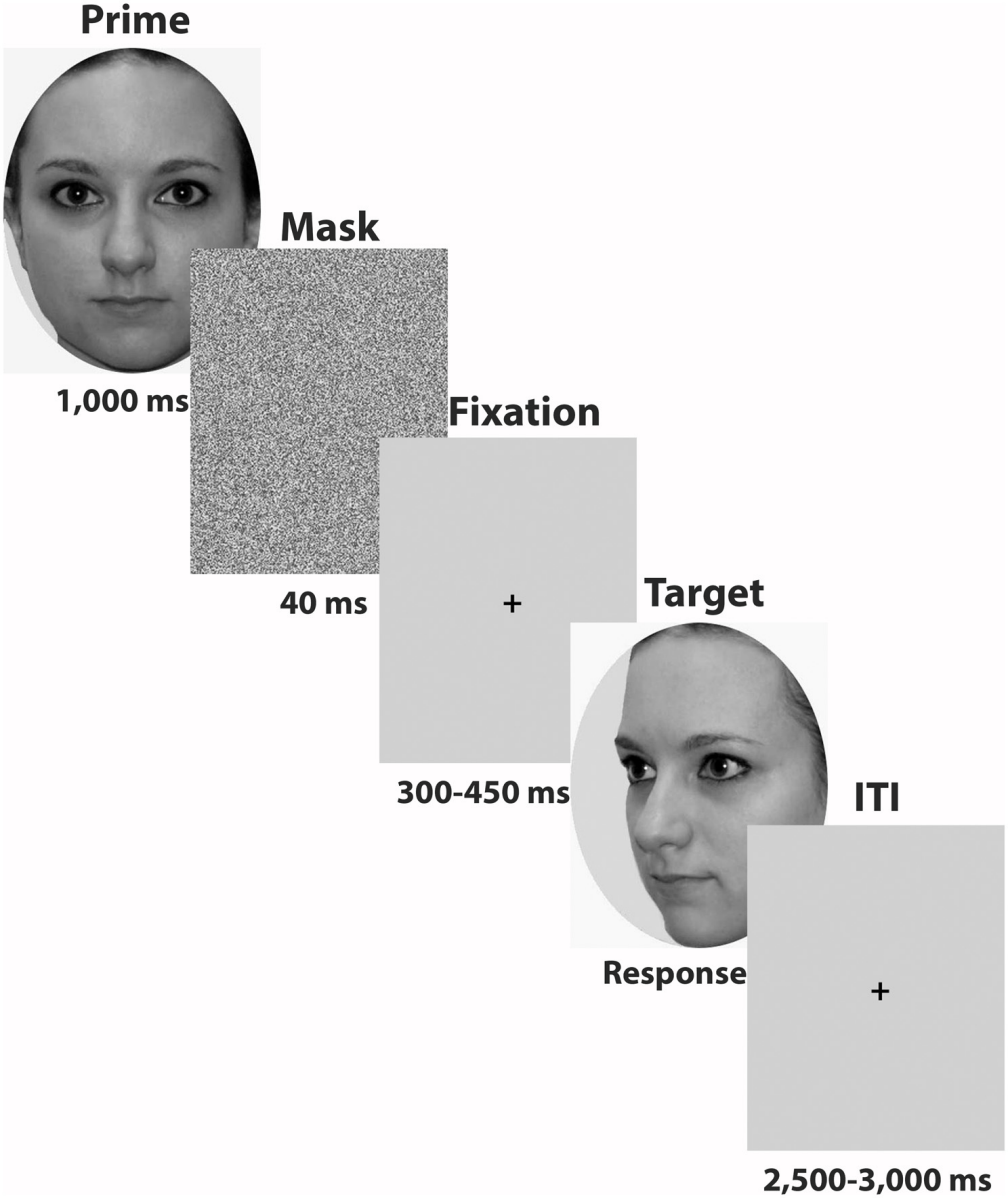
Example of a congruent trial, with the target face shown in three-quarter view.

Participants ran 20 practice trials followed by three blocks of test trials. A trial comprised of the presentation of a prime face (frontal view; friend, famous, unfamiliar) for 1 s, followed immediately by a white noise mask presented for 40 ms. This was followed by a fixation cross, for an ISI of between 300–450 ms (varying pseudo-randomly, with an even distribution across trials). A target face image then appeared on the screen, and remained until the participant responded. The target face presented was either of the same identity as the prime face (congruent) or of a different identity (incongruent). The target face was presented either in frontal view, three-quarter view or profile view (see Fig 1; Please note that the individual pictured in Fig 1 has given written informed consent—as outlined in PLOS consent form—to publish this image). Using three fingers on one hand, participants were required to press a button on the keyboard (“c”, “v” or “b”) to indicate whether the target face was their friend’s face, a famous face or an unfamiliar face, and were instructed to respond as quickly and as accurately as possible. The order of the buttons allocated to “friend”, “famous” and “unfamiliar” was counterbalanced across participants. Each trial was followed by an inter-trial interval (ITI) varying between 2,500 and 3,000 ms (varying pseudo-randomly, with an even distribution across trials).

Trials were balanced such that each prime face identity (friend, famous, unfamiliar) was paired with a congruent or incongruent target face identity an equal number of times. Each target face was presented an equal number of times in the frontal, three-quarters and profile view. Trials were presented in randomised order. Each testing block comprised 216 trials (3 target face identities x 3 view types x 2 prime-target congruencies x 12 repetitions each).

## Results

As expected with a simple target identification task, accuracy performance was at ceiling level (mean = 98.01%, SD = 2.02), and was not analysed further. For each participant, RT’s more than two standard deviations away from that participant’s mean were removed as outliers [23]. Data can be found at 10.6084/m9.figshare.2061291. Reaction time for correct responses were analysed using a 2 x 3 x 3 repeated-measures ANOVA, with IVs of Prime-Target Congruence (congruent, incongruent), Target Face Identity (friend, famous, unfamiliar), and Target View (frontal, three quarter, profile). All post-hoc tests were interpreted using Bonferroni adjustment for multiple comparisons.

Analyses showed a significant effect of Prime-Target Congruence, *F*(1, 39) = 40.43, *p* < .001, ƞ_p_^2^ = .509, with participants demonstrating a reliable priming effect whereby they responded more quickly to target faces of the same identity as the prime face (673.26 ms, *SE* = 19.37) compared to when prime-target face identity was incongruent (708.57 ms, *SE* = 18.38). A significant effect of Target View was also observed, *F*(2, 78) = 4.34, *p* < .05, ƞ_p_^2^ = .100. Overall, participants responded more quickly to target faces when they were presented in frontal view compared to profile view, *t*(39) = 2.90, *p* < .017. There was no difference between faces presented in frontal and three-quarter views, *t*(39) = 1.49, ns., or between faces presented in three-quarter and profile views *t*(39) = 1.50, ns.

**Fig 2:**
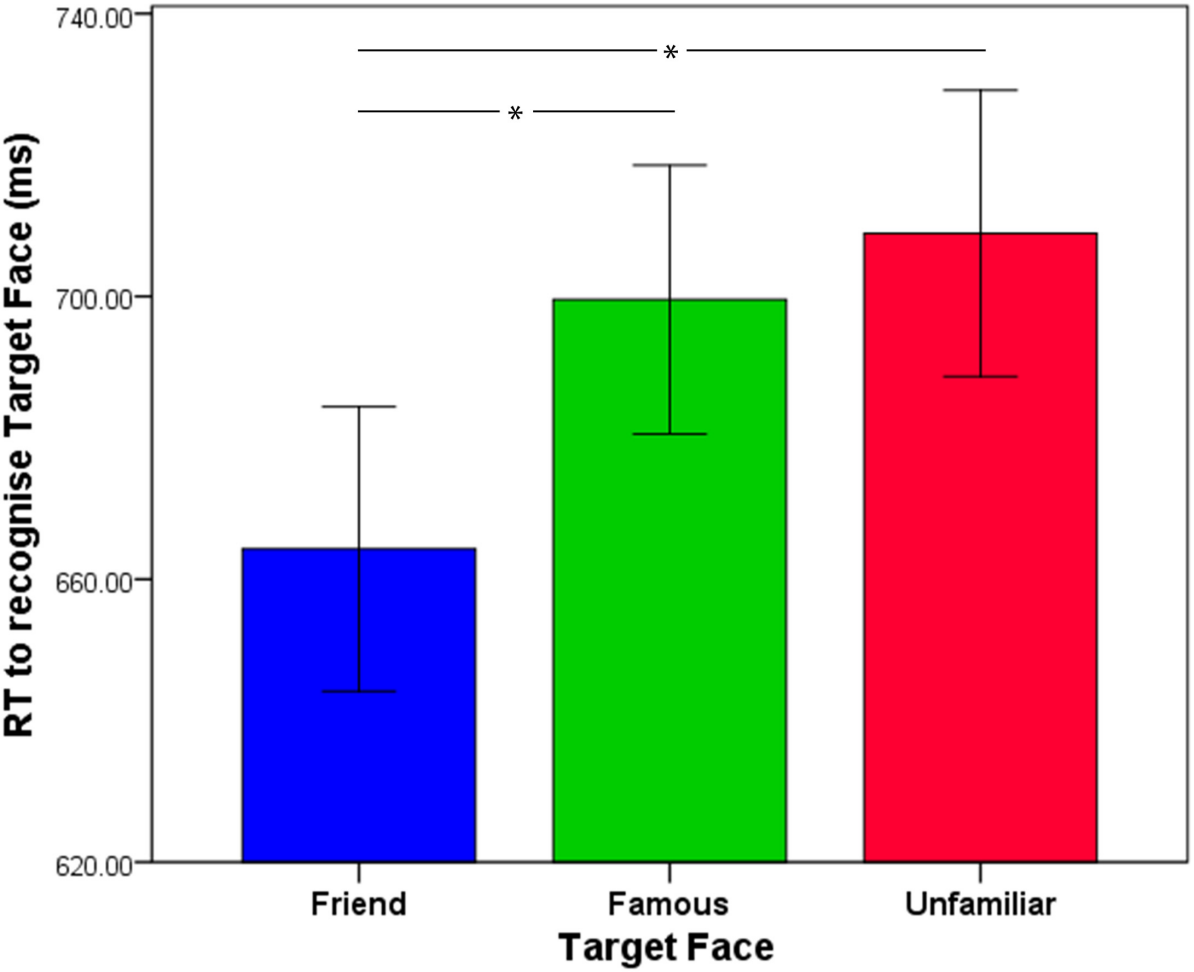
Response times to a friend’s face, famous face and unfamiliar face. Mean reaction times for correct responses to recognise a target friend’s face (blue) famous face (green) and unfamiliar face (red). Error bars represent the standard error of the mean.

Most interestingly, a significant effect of Target Face Identity was observed, *F*(2, 78) = 8.47, *p* < .001, ƞ_p_^2^ = .178, with participants responding significantly more quickly to friend faces compared to either famous faces, *t*(39) = 2.95, *p* < .017, or unfamiliar faces, *t*(39) = 3.41, *p* < .017. There was no difference in response time to recognising famous and unfamiliar faces, *t*(39) = 1.06, ns. (see Fig 2).

**Fig 3:**
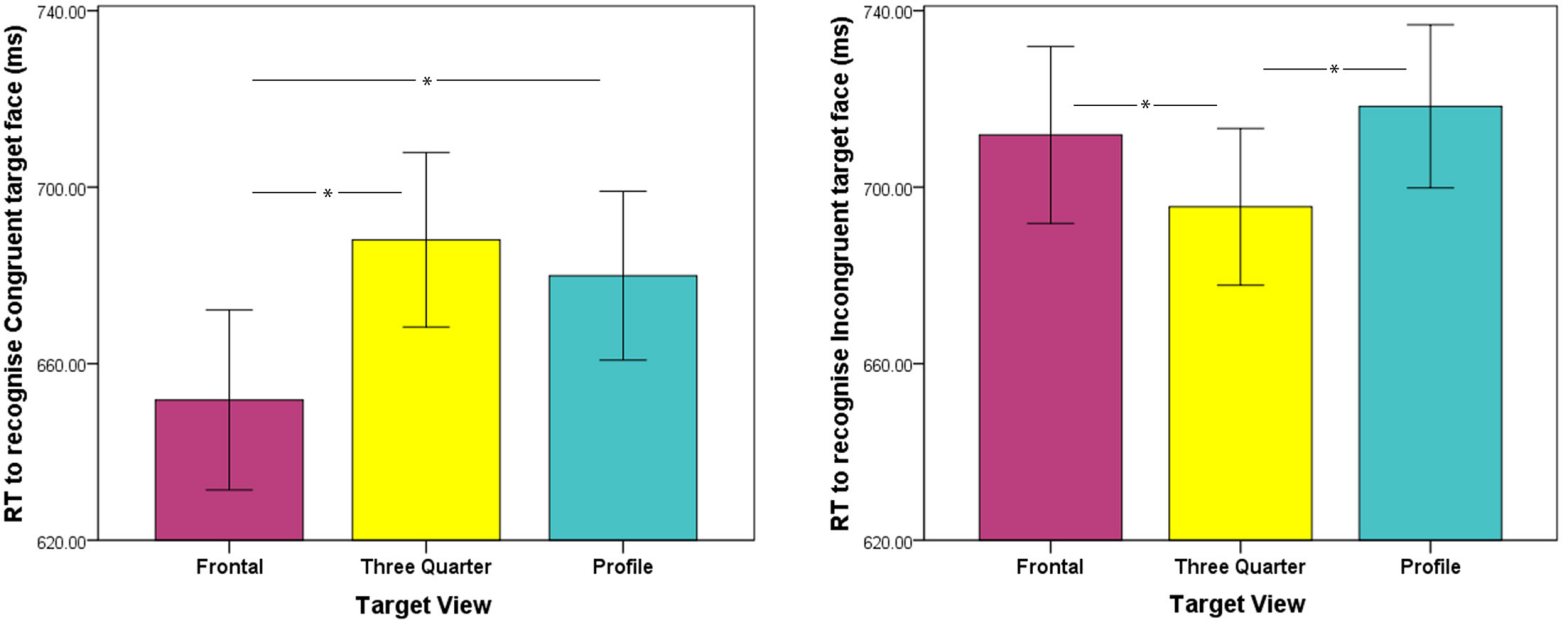
Response times to congruent and incongruent trials across viewpoints. Mean reaction times for correct responses to recognise target faces presented in frontal view (purple), three quarter view (yellow) and profile view (teal) for congruent (left panel) and incongruent (right panel) trials. Error bars represent the standard error of the mean.

Finally, a significant interaction between Prime-Target Congruence and Target View, *F*(2, 78) = 26.48, *p* < .001, ƞ_p_^2^ = .404, revealed a significant simple main effect for congruent trials, *F*(2, 78) = 14.47, *p* < .001, ƞ_p_^2^ = .271, such that participants responded significantly more quickly to frontal target views compared to either three-quarter, *t*(39) = 4.69, *p* < .017, or profile views, *t*(39) = 3.86, *p* < .017. This suggests that for congruent trials, participants’ responses were driven by pictorial cues, as prime pictures were always presented in frontal view. There was no difference in response time to profile and three-quarter views for congruent trials, *t*(39) = 1.37, ns. For incongruent trials, a different pattern emerged. Here, a significant simple main effect, *F*(2, 78) = 5.98, *p* < .005, ƞ_p_^2^ = .133, revealed that responses to three-quarter view faces were faster than responses to either frontal, *t*(39) = 2.14, *p* = .019, or profile views, *t*(39) = 4.03, *p* < .017. There was no difference in response time for frontal and profile views, *t*(39) = 0.93, ns. This suggests that, when Prime-Target Identities did not match, participants relied on stored representational cues in order to make an identity decision (see Fig 3).

No three-way interaction between Prime-Target Congruence, Target Face Identity and Target View was observed *F*(4, 156) = 0.70, ns., ƞ_p_^2^ = .018, nor did Target Face Identity interact with either Prime-Target Congruence, *F*(2, 78) = 1.02, ns., ƞ_p_^2^ = .026 (S1 Fig), or Target View, *F*(4, 156) = 1.51, ns., ƞ_p_^2^ = .201 (S2 Fig).

## Discussion

This study looked at whether socially important faces would benefit differentially from attentional priming, relative to other familiar and unfamiliar faces. While a reliable priming effect was established overall, this did not affect socially important (personally familiar), famous and unfamiliar faces differently. Most interestingly, a “social importance” effect was observed, whereby processing was speeded for socially important faces relative to either famous faces or unfamiliar faces. Processing speeds for famous and unfamiliar faces did not differ. Finally, changes in viewpoint between prime and target faces did not affect socially important, famous and unfamiliar faces differently. These effects are discussed in detail below.

### Personally familiar versus famous face processing

A main finding of this paper is that responses to socially important personally familiar faces are consistently faster than responses to either unfamiliar *or famous* faces. This speeded processing may reflect a “social importance” effect, whereby we are tuned to respond preferentially to stimuli which are socially relevant to us. Speeded responses to socially important personally familiar faces—but not to famous faces—suggests that the observed results did not simply reflect a familiarity effect. Our finding of no difference in processing speed when participants were responding to socially unimportant famous and unfamiliar faces was unexpected, as several studies which directly compare famous and unfamiliar face processing find an advantage for famous faces [24–25], [5]. We suggest that when participants are presented with a paradigm which includes familiar famous faces, personally familiar faces and unfamiliar faces, they primarily make classification decisions based on the social importance of the face, rather than mere familiarity. That is, socially important faces are prioritised over socially unimportant (famous and unfamiliar) faces. In situations where only famous and unfamiliar faces are compared [24–25], [5], we may then revert to judgements based primarily on familiarity.

To our knowledge, only one previous study has shown speeded reaction time performance to personally familiar faces, compared to either famous or unfamiliar faces [16]. Our study demonstrates that this “social importance” effect occurs when attention is both primed and unprimed, and is independent of changes to viewpoint. That is, we report a robust effect of preferential processing for socially important faces when included in a paradigm with socially unimportant familiar faces and unfamiliar faces. One implication of this finding is that studies investigating the effects of familiarity in face processing should take into consideration the social importance of the face. Indeed, studying familiarity using personally familiar faces (rather than famous faces) may be advisable, as this may be tapping into a more naturalistic type of “familiarity” judgement, tied to social importance.

Using a priming paradigm allowed us to investigate whether any advantages found for personally familiar face processing were due to *attentional* effects [1,2]. Here we report that personally familiar faces did not selectively grab attention (i.e. priming was not more effective for friend faces compared to famous or unfamiliar faces). We conclude that the preferential processing invoked by personally familiar faces is not likely to be tied to an attentional effect, but rather reflects a speed of processing advantage for these important types of face. This suggests that personally familiar faces may be more robustly represented than other types of familiar or unfamiliar faces [11].

It remains a possibility that speeded processing of personally familiar faces compared to famous faces occurs as a result of the amount or quality of exposure to these different types of face. In conducting a pilot study to ensure that the famous faces used were extremely recognisable, and having individual participants confirm that the famous faces used in their trials were indeed very well known to them, we were able to establish that participants were highly familiar with both types of familiar face (friend, famous). However, it is likely that participants would have exposure across a greater range of viewpoints and conditions for personally familiar compared to famous faces, and this may also have contributed to the effect. That we found the effect to be independent of viewpoint, however, strengthens the suggestion that the processing advantage for personally familiar faces may result from preferential processing due to their social importance.

### Viewpoint Dependence

Surprisingly, viewpoint changes between prime and target faces did not affect familiar and unfamiliar faces differentially. Several studies have shown that it is easier to match familiar faces across viewpoints compared to unfamiliar faces [26–29], possibly due to a more robust—and therefore more view-independent—representation of highly familiar faces. In this experiment, participants did not demonstrate stronger viewpoint-dependence for unfamiliar faces, as predicted. Indeed, no difference was observed between personally familiar, famous and unfamiliar faces in terms of a reliance on viewpoint-dependent cues. We infer that for a simple priming task, participants were able to generalise across viewpoints for all classes of face. It is certainly possible to recognise an unfamiliar face across different viewpoints, even after a single viewing [30–32], and our study supports this finding. That images of unfamiliar faces were no more viewpoint-dependent than images of personally familiar or famous faces suggests that rapid learning and generalisation across viewpoints took place.

An alternative explanation could relate to the relatively long prime duration (1,000 ms) used in our experiment. Indeed, Huber and O’Reilly [33] suggest that for repetitive priming tasks at least, effects are strongest when the prime is presented for < 400 ms, diminishing in size until 2,500 ms. Others report that, while significant priming effects can be observed for prime durations of both 250 ms and 2,000 ms, priming is more effective in the shorter duration condition [34]. However, studies using similar prime durations to the one used in this experiment report significant priming effects (e.g. [35]), and Neely reports that reliable priming effects are observed at up to 2,000 ms SOA [36]. That we found a robust main effect of priming in our study suggests that our prime duration was effective in priming target responses. It remains possible that more nuanced priming effects on identity could be drawn out using shorter prime durations, and this merits further investigation. Furthermore, that the primes were task-irrelevant may have implications for the interpretation of our results. While task-irrelevant priming does certainly occur (e.g. [37]), and we did observe strong priming effects in our experiment, perhaps a task which asked participants to directly compare prime and target faces would elicit more graduated responses—this remains a topic for study.

While participants responded more quickly to frontal compared to profile views of all types of face, a different pattern of results was observed for identity congruent and incongruent trials. It appears that for congruent trials, participants relied on pictorial cues when responding to a target face; that is, when a prime face (always frontal view) preceded an identity-congruent target face, participants responded faster when the target face view matched the prime face view. This supports recognition accuracy data produced by Logie and colleagues [18]. In contrast, when the prime face identity was incongruent to the target face, participants appeared to rely more heavily on stored representations of the faces. This was evidenced by speeded responses to target faces presented in the three-quarter view. We gain most information from a face presented at an angled view [38–40], and it is likely that faces presented in the three quarter view provided most information for participants to match with their stored representations.

## Conclusion

Results from this paper suggest that socially important personally familiar faces are processed preferentially to socially unimportant familiar and unfamiliar faces. Indeed, when presented in a context which includes socially important personally familiar faces, famous faces are processed at the same speed as unfamiliar faces. We recommend that studies of social attention, as well as other studies of the effects of familiarity in face perception, take into account the special nature of socially important stimuli. The social importance of a face may play an important role in recruiting preferential processing.

## Supporting Information

S1 Fig Response times to Target Face Identity for congruent and incongruent trials. Mean reaction times for correct responses to recognise friend (blue), famous (green) and unfamiliar (red) target faces for congruent and incongruent trials. Error bars represent the standard error of the mean.
(TIF)

S2 Fig Response times to Target Face Identity across viewpoints. Mean reaction times for correct responses to recognise friend (blue), famous (green) and unfamiliar (red) target faces at frontal, three quarters and profile views. Error bars represent the standard error of the mean.
(TIF)
