# Supplementary figures and images for: Socially Important Faces Are Processed Preferentially to Other Familiar and Unfamiliar Faces in a Priming Task across a Range of Viewpoints
Source: PLoS One. 2016 May 24;11(5):e0156350. doi: 10.1371/journal.pone.0156350 (PMC4878734; doi:10.1371/journal.pone.0156350)

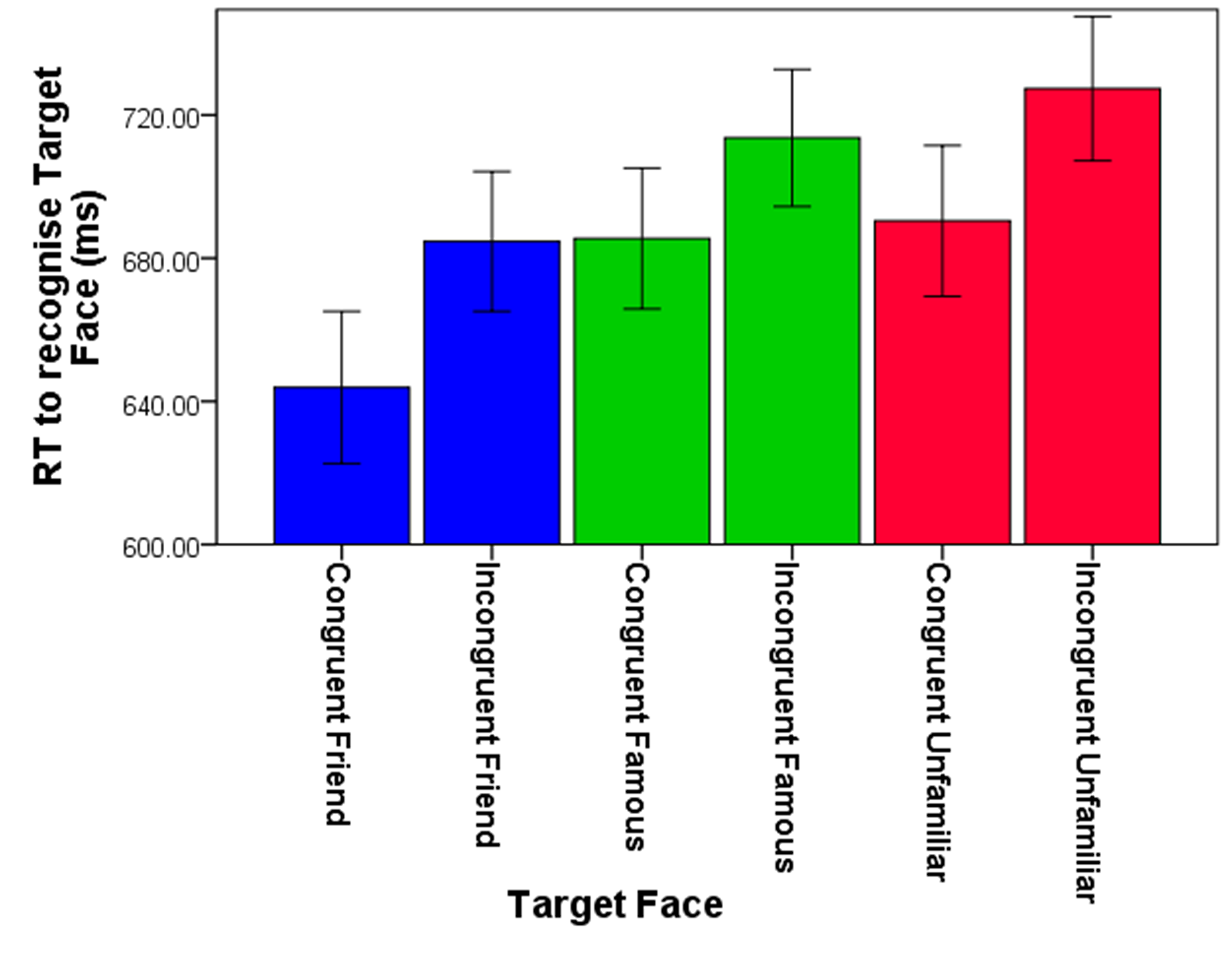

Supplement: S1 Fig — Mean reaction times for correct responses to recognise friend (blue), famous (green) and unfamiliar (red) target faces for congruent and incongruent trials. Error bars represent the standard error of the mean. (TIF) [file pone.0156350.s001.tif]

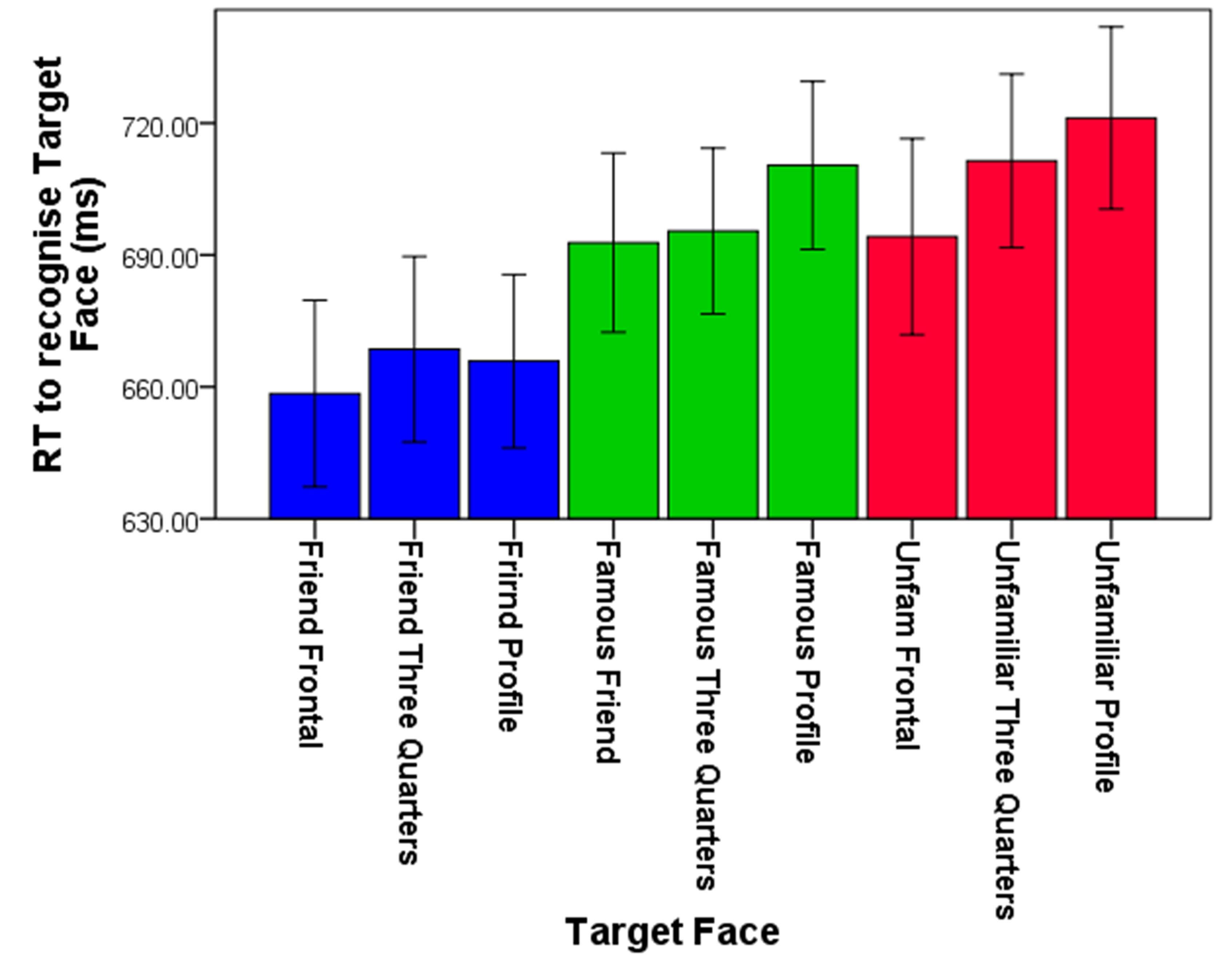

Supplement: S2 Fig — Mean reaction times for correct responses to recognise friend (blue), famous (green) and unfamiliar (red) target faces at frontal, three quarters and profile views. Error bars represent the standard error of the mean. (TIF) [file pone.0156350.s002.tif]
